# Genetic Evaluation of Patients With Delayed Puberty and Congenital Hypogonadotropic Hypogonadism: Is it Worthy of Consideration?

**DOI:** 10.3389/fendo.2020.00253

**Published:** 2020-05-19

**Authors:** Adalgisa Festa, Giuseppina Rosaria Umano, Emanuele Miraglia del Giudice, Anna Grandone

**Affiliations:** Department of Woman, Child, General and Specialized Surgery, University of Campania L. Vanvitelli, Naples, Italy

**Keywords:** puberty, genetics, hypogonadal hypogonadism, next-generation sequencing, pediatrics

## Abstract

Delayed puberty is a common reason of pediatric endocrinological consultation. It is often a self-limited (or constitutional) condition with a strong familial basis. The type of inheritance is variable but most commonly autosomal dominant. Despite this strong genetic determinant, mutations in genes implicated in the regulation of hypothalamic–pituitary–gonadal axis have rarely been identified in cases of self-limited delayed puberty and often in relatives of patients with congenital hypogonadotropic hypogonadism (i.e., *FGFR1* and *GNRHR* genes). However, recently, next-generation sequencing analysis has led to the discovery of new genes (i.e., *IGSF10, HS6ST1, FTO*, and *EAP1*) that are implicated in determining isolated self-limited delayed puberty in some families. Despite the heterogeneity of genetic defects resulting in delayed puberty, genetic testing may become a useful diagnostic tool for the correct classification and management of patients with delayed puberty. This article will discuss the benefits and the limitations of genetic testing execution in cases of delayed puberty.

## Definition

Delayed puberty (DP) is defined as the lack of pubertal signs, namely, thelarche in females and increase of testicular volume (≥4 ml) in males, at an age above 2–2.5 standard deviation of the population mean classically set at 13 years for girls and 14 years for males or a stunted pubertal progression diverging from puberty nomograms (1). DP is a frequent condition, affecting 2% of subjects in pubertal age.

## Etiology and Epidemiology

In line with two large clinical reports on this topic (2, 3), DP can result from different conditions. Constitutional delay of growth and puberty (CDGP) is the most frequent cause (4), characterized by growth retardation in childhood and delayed puberty in adolescence, also termed as self-limited DP, which generally spontaneously reaches completion by 18 years of age. Self-limited DP is found in 73% of boys and in 43% of girls with pubertal delay (2, 3).

Hypergonadotropic hypogonadism is an expression of gonadal failure. It affects especially females where it represents 21% of DP cases, with Turner syndrome in 27% of the affected girls (2, 3).

Another condition of DP is central hypogonadotropic hypogonadism, which can be distinguished in functional hypogonadism in case of chronic disease and nutritional or stressing factors able to inhibit the activation of the hypothalamic–pituitary–gonadal (HPG) axis, corresponding to approximately 16–20% of DP (2, 3), and permanent hypogonadism, affecting 15% of females and 8% of males. Permanent hypogonadism can be congenital (CHH), both isolated and syndromic, or acquired. In particular, CHH offers a wide spectrum of clinical manifestations ranging from severe forms with clinical signs of neonatal gonadotropin deficiency through milder forms of pubertal development arrest to reversible forms of hypogonadism. CHH accounts for 4% of boys and 5.7% for girls with DP (2, 3).

The clinical challenge is the differentiation between self-limited DP and other forms of hypogonadism, especially hypogonadotropic hypogonadism. This review aims to summarize the knowledge about the genetic etiology of both CHH and self-limited DP to suggest a possible role for genetic testing in clinical setting.

## Differential Diagnosis

In clinical setting, it is useful to identify “red flags” in medical history or clinical examination that suggest the possible etiology of DP (5). A family history of delayed puberty with an autosomal dominant inheritance emerging from pedigree could indicate both self-limited DP and CHH.

In male newborns, the finding of micropenis and cryptorchidism is crucial for the suspicion of permanent hypogonadism. Indeed in Kallmann syndrome (KS), micropenis was described in 20 to 40% (6–8) of newborns, while cryptorchidism affected 30 to 50% of CHH males (9, 10). Minipuberty offers an important window for evaluation of the HPG axis. In this phase, hormonal assessment, including gonadotropins and sex steroids, can allow to identify CHH based on the lack of physiological hormonal surge. In females, there are no equivalent clinical signs for suspicion of CHH. In both sexes, in the presence of one of the parents with CHH, hormonal evaluation during minipuberty is recommended and has to be completed by genetic testing in case of a known mutation in the affected family member (11). In medical history, it is important to investigate the signs or symptoms of impaired thyroid function (hypothyroidism or hyperthyroidism), of chronic diseases like inflammatory bowel disease or coeliac disease and to evaluate pathological food dynamics with food restriction and excessive physical activity suggesting anorexia nervosa. Moreover, attention should be paid to a chronic use of corticosteroids and a positive history of hematological diseases with a chronic transfusion regime determining hemosiderosis. In patients with a history of chemotherapy or radiation therapy, it is possible to hypothesize both gonadal failure and hypogonadotropic hypogonadism. Recent onset of diplopia, headache, vomiting, and seizures could suggest a brain mass.

Similarly, a detailed clinical exam can reveal important elements for diagnostic workup. Attention should be given to the presence of middle line defects or typical facial features, visual impairment, deafness, bimanual synkinesia, anomalies of hand or foot, dental or skull dysgenesis or malformations and complex malformation association suggesting a CHARGE syndrome. Conversely, anosmia or hyposmia, as evaluated by olfactometry, offers a strong suspicion of KS.

At initial presentation, a differential diagnosis between self-limited DP and CHH is difficult because patients have common clinical and biochemical characteristics. Another important aspect is the possible reversal of hypogonadism, to date described in patients carrying variations in nine genes (*KAL1, FGFR1, CHD7, HS6ST1, PROKR2, NSMF, GNRHR, TAC3* and *TACR3*) (11), that complicates the differential diagnosis between CHH and self-limited DP in cases with a mild normosmic phenotype.

## Genetics of CHH

The current knowledge about pubertal delay pathophysiology is mainly derived from defects in genes determining CHH. CHH represents from 24 to 85% of permanent hypogonadotropic hypogonadism and includes two subgroups of patients: normosmic subjects (nCHH) and subjects with anosmia and other clinical signs of KS like deafness, cleft lip/palate, renal anomalies and synkinesis, representing 50% of cases of CHH. Anosmia reflects the involvement of genes that play a role in the development of GnRH neurons and olfactory bulbs, while in nCHH mutations affect the genes involved in GnRH secretion or function. Nevertheless, the usefulness of this dichotomous distinction in targeting genetic testing is limited by the clinical overlap of the two conditions (12).

In CHH, a genetic cause is found in about 50% of patients (13, 14). To date, mutations in more than 30 genes have been identified as a genetic cause of CHH, both nCHH and KS, with some rare loci involved in complex syndromes (11, 15–18). As for several other diseases, the application of next-generation sequencing (NGS) technology to CHH diagnosis has been responsible for the identification of an increasing number of genes involved in its etiology.

In fact, the genetic heterogeneity and the overlapping phenotypes make CHH the classical disease in which new next-generation sequencing technology finds application.

The inheritance pattern in CHH includes different modes, namely, autosomal dominant, autosomal recessive and X-linked. In addition, *de novo* mutations have been reported, whereas for some genes incomplete penetrance and variable clinical expressivity are frequent.

## Autosomal Recessive Forms

Among the autosomal recessive forms, the most frequent findings are biallelic mutations in three genes: *GNRHR*, for which more than 60 families have been described (11, 19–24), *KISS1R*, with 27 families identified (19, 25, 26), and *TACR3*, found in 20 families (27–31). In these cases, the phenotype is a classical nCHH, without any non-reproductive signs of pathology (16). Interestingly, for *GNRHR* variations, that are responsible of about 40–50% of inherited nCHH cases (23), there is a wide phenotypic variability even in the same family with the same genetic variant (32). Another important aspect is the possible reversal of *TACR3* and *TAC3* mutation-related hypogonadism. In these cases, the differential diagnosis between CHH and self-limited DP can be supported by the possible presence of micropenis that suggests *TACR3* mutation (30). On the other hand, biallelic mutations in genes encoding for ligands of the above-mentioned receptors, like *GNRH1, KISS1*, and *TAC3*, are a rare cause of nCHH; even rarer are those in *LHB* and *FSHB* (16).

In this group, we find examples of genes involved in the neuroendocrine regulation of GnRH neurons. *GNRHR* encodes for a G-protein-coupled receptor that, through a variation of intracellular calcium levels, determines the pituitary release of gonadotropins (33); therefore, *GNRHR* mutations represent a paradigm of altered function of GnRH. In addition, *KISS1R, TAC3*, and *TACR3* encoding for a G-protein-coupled receptor for Kisspeptin, for neurokinin B and neurokinin receptor, respectively, are all part of a complex network. In this network, the KDNY neurons in arcuate nucleus that synthesize kisspeptin, neurokinin B, and Dynorphin exert a regulatory role on GnRH neuronal function (17).

## X-Linked Forms

*ANOS1*, previously *KAL1*, located on Xp22.3, encodes for Anosmin1, an extracellular protein mediating cellular adhesion, playing a fundamental role in the migration process of olfactory and GnRH neurons that leads these cells from nasal placode to the hypothalamus (33). *ANOS1* is characterized by an X-linked recessive pattern of inheritance; mutations or intragenic microdeletions of this gene are responsible for 10–20% of KS (13). The penetrance is complete for typical clinical manifestations, such as CHH and anosmia (34–39). Conversely, other clinical manifestations like synkinesis, revealed in 75% of patients (34), and renal agenesis, found in 30% of subjects, display different expressions in individuals carrying the same variant (35–46). To date, about 144 families have been described. Among them, an interesting finding was a female phenotype in 10 subjects, of whom one case was due to a biallelic *ANOS1* mutation (47) and the remaining nine cases were due to a second variant in another gene, suggesting a possible oligogenic mechanism.

Among genes with X-linked transmission, defects in *DAX1* determine a syndromic association between CHH and congenital adrenal hypoplasia; although penetrance is near-complete (48, 49), phenotypic variability is seen both in the severity of CHH (48–52) and in the age at onset of adrenal insufficiency (48–50, 53, 54).

## Autosomal Dominant Forms

Among autosomal dominant forms of CHH, including nCHH and KS, two genes are the most frequently involved, *FGFR1* and *CHD7*.

*FGFR-1* encodes for a tyrosin kinase receptor able to activate a complex signaling, including also *ANOS1* and *FGF8*, regulating fundamental developmental processes like neuronal migration, fate, cell survival and proliferation (33). *FGFR1* plays an important role, with more than 140 mutations described, generally determining a loss of function with several mechanisms (nonsense, missense, frameshift, splicing and rarely deletions) (34, 55, 56). *De novo* mutations are another relatively frequent possibility (11, 35). The *FGFR1* mutations determining KS are characterized by incomplete penetrance (11, 57, 58) and variable clinical expression of the same mutation in the same family, with patients displaying complete phenotype, only anosmia, or isolated pubertal delay (57–62). Furthermore, as reported by various authors, mutations in this gene cause also nCHH (58, 63–67). Other clinical features of *FGFR1* mutations, such as skeletal anomalies, cleft lip and cleft palate and dental agenesis, are present with variable frequency (11, 34, 44, 57, 58, 60, 61).

*CHD7* gene, located in 8q12.1, is a well-known genetic cause of CHARGE syndrome, characterized by coloboma, heart anomalies, choanal atresia, growth and development retardation, genital and ear anomalies. Subsequently, it was recognized as a genetic cause of nCHH and KS (68–71). In these cases, genetic variants consist in missense mutation with a partial loss of function (70, 71). Furthermore, *de novo* mutations are frequently found (69, 70). As in the case of *FGFR1* mutations, there is wide phenotypic variability, ranging from KS through nCHH to isolated anosmia (70, 71). Other clinical manifestations associated with *CHD7* mutations in patients with CHH are deafness, anomalies of the outer ear and lip/cleft palate (70–73). The expression of chromodomain-helicase-DNA-binding protein 7 (*CHD7*) in the hypothalamus and in the olfactory epithelium reflects a possible role in the development of olfactory bulb and GnRH neurons (33).

Recently, autosomal dominant mutations in *SOX10* were described as a cause of KS (74–79), these patients also presented neurogenic deafness. The SOX transcription factor family is involved in the development of a large number of organs, in particular, *SOX10* is expressed in GnRH cell precursors (16).

In this section, *PROKR2*, a gene that encodes for a G-protein-coupled receptor, and *PROK2*, encoding for prokinecitin 2, a ligand of this receptor, should be mentioned. The ligand binding to the receptor triggers a signaling cascade with effects on the development of the olfactory system and the progenitors of GnRH neurons (33). *PROKR2* mutations are found both in KS and nCHH. This gene is characterized by autosomal recessive inheritance pattern in 20% of cases; the remaining cases are due to autosomal dominant or oligogenic mechanisms with the involvement of other genes (80–88). Of note is that some *PROKR2* rare variants are present in the general population with a significant prevalence. Therefore, it is difficult to interpret the genetic results, also considering the incomplete penetrance, when analyzing a pedigree. The *PROK2* variants instead are rare and can present with autosomal dominant or recessive pattern of inheritance.

Another interesting gene is *FGF8*, which encodes for a ligand of *FGFR1* and therefore taking part in this signaling pathway (16). Heterozygous mutations were found in KS and nCHH, also with reported cases of oligogenic inheritance. The clinical manifestations include neurosensorial deafness, cleft lip/palate and camptodactyly (11, 89–91).

## Oligogenic Inheritance

Recently, a mutation in two or more genes, with an oligogenic inheritance, was reported in several cases of nCHH and KS. The first description was in 2006 in a case of KS due to *PROKR2* and *KAL1* mutations (80). In 2010, Sykiotis et al. (92) analyzed a large series of CHH patients, finding oligogenic inheritance in 2.5% of the subjects. Later, other groups reported an oligogenic mechanism in 7% (67) to 15% (12) of subjects with CHH. To date, at least 16 genes are known to contribute to oligogenicity (11). The application of next-generation sequencing increases the chance to find oligogenism in CHH. In some cases, distinction between oligogenism and the presence of benign variants not interfering with the phenotype could be challenging (16).

Gene defects underlying CHH concern genes encoding for proteins involved in different fundamental physiological mechanisms: development and migration of GnRH neurons as in the case of *ANOS1, FGFR1, FGF8, CHD7, PROK2*, and *PROKR2*; the regulation of GnRH secretion as in the case of *KISS1R, TACR3* and *TAC3*; GnRH action as in the case of *GNRHR*.

### Genetic Analysis in CHH

Until the introduction of NGS, the genetic diagnosis in CHH was obtained mainly through Sanger sequencing, analyzing gene exons and exon–intron junctions one by one. This analysis could now represent an appropriate choice in case of a patient belonging to a family with a known mutation that completely explains the observed phenotype, with fast and cost-effective results. Conversely, in the case of an initial genetic evaluation of a patient with CHH, given the large number of genes involved in this condition and the phenotype heterogeneity, Sanger sequencing could result to be time-consuming and expensive.

The growing diffusion of NGS offers the possibility of simultaneous analysis of many genes, resulting in a time-sparing and cost-effective strategy (16).

This approach can expand the phenotype of known genetic diseases through the genetic diagnosis in patients with incomplete or atypical clinical manifestations and, conversely, in the case of whole-exome or whole-genome sequencing, can reveal new genes that are responsible of a genetic disease.

An important limitation is the enormous mass of data derived from this type of analysis, in which a careful and adequate choice of pipelines and bioinformatic filters to improve the detection rate and the interpretation of results plays a fundamental role. Beyond these technical aspects, a complete phenotypical description of the case, with a precise assessment of the familial pedigree and of the hypothetical mode of transmission is needed for the attribution of clinical validity to an identified variant (16).

NGS techniques include whole-exome sequencing (WES) target sequencing (TS) and whole-genome sequencing (WGS). WES and target-exome sequencing evaluate only exons of all genes and of a panel of genes selected for their known role in the disease, respectively, without sequencing intronic and potential regulatory regions and furthermore with a coverage of exon-intron junction varying on the basis of technical design characteristics. The WGS meanwhile consists in sequencing of the entire genome, including also intronic regions, with the possibility to find variants in intronic and intergenetic regions. The cost of this latter technique is still high; furthermore, a couple of limitations of WGS may be the lower depth of sequence coverage compared to WES and difficult interpretation.

Finally, in a patient with CHH and a complex phenotype in which a possible genetic explanation could be a contiguous gene syndrome, array-CGH can be appropriate to exclude submicroscopic copy number variation (microdeletion and microduplication) (17).

In line with costs and with potential genetic relevance of information derived from a genetic analysis in the field of CHH, considering the known genetic heterogeneity of CHH and the possibility of oligogenicity, WES or target sequencing (depending also on local facilities) appears to be the test of choice among NGS techniques in clinical settings (16).

However, the next-generation sequencing represents an advantageous technique, but the interpretation of the results could be challenging, so a detailed phenotypic characterization is very important.

### Importance of Genetic Diagnosis in CHH

A genetic diagnosis represents the conclusion of the diagnostic path, with implication on prognosis, in particular in view of the possibility of reverse, on correct counseling for other family members and also for the patient's offspring. Genetic counseling starts from the evaluation of the mode of inheritance according to pedigree and gene defect found. The counseling becomes more complex in case of oligogenism, indeed to establish the role of each variant requires a deep knowledge of the phenotype related to the single variant and the availability of informative pedigree composed by affected and unaffected subjects (16).

### Overlapping Etiology Between CHH and Self-Limited Delayed Puberty

Considering the distribution of puberty timing, self-limited DP can be assimilated to the extreme upper limit of normality. On the other hand, the frequent presence of a family history of pubertal delay induced to suppose a genetic basis for this condition, with an apparent autosomal dominant inheritance.

A study (93) based on record review and interviews analyzed the pedigrees of 53 subjects with CDGP and 25 controls, finding an apparent autosomal dominant pattern of inheritance in the majority of families. Moreover, they reported an increased risk for pubertal delay in the relatives of subjects with CDGP (RR 4.8 and 3.2 for first degree and for second degree relatives, respectively) compared to controls.

The identification of the genetic cause in self-limited DP offers various pitfalls because DP is a common condition in non-affected individuals. Therefore, a genetic variant possibly determining this condition could have a quite relatively high prevalence in the general population.

Moreover, the presence of pubertal delay in 10% of relatives of CHH patients (94) and the possibility of a spontaneous reversal in 10% of patients with CHH (12) induce to hypothesize a shared molecular basis in CHH and self-limited DP.

In the general population, the timing of puberty is influenced by general health, nutritional status and endocrine disruptors chemicals but is under a strong genetic influence (95–98). The knowledge about the genetic control of the hypothalamic–pituitary–gonadal axis derives mainly from studies on subjects with GnRH deficiency, leading to the discovery of rare variants underlying CHH. Different studies were conducted to explore the role of genes causing CHH in determining self-limited DP.

In their work, Zhu et al. (94) examined the hypothesis of a shared genetic basis between these two conditions using WES in two different cohorts. They analyzed 15 pedigrees with an IHH proband carrying a potentially pathogenic variant in IHH genes (*FGF8, FGFR1, GNRH1, HS6ST1, KAL1, KISS1, KISS1R, NELF, PROK2, PROKR2, TAC3*, and *TACR3*) and family members both with delayed and with normal puberty. A genetic variant was found in 53% of relatives with DP and in 12% of relatives with normal puberty. In the other cohort of 56 DP subjects with no family history of IHH matched with controls from ExAC, they found potentially pathogenic variants in IHH genes in 14.3% of DP subjects and in 5.6% of controls. The heterozygous potentially pathogenic variants were in *GNRHR, TAC3, TACR3, SEMA3A* and *IL17RD*, the latter with the largest number of subjects. However, the controls also carried potentially pathogenic variants. An important observation is that incomplete penetrance and variable clinical expressivity represent a hurdle for which genetic testing in differential diagnosis between IHH and self-limited DP might have limited usefulness.

Also, Cassatella et al. (12) tried to explore the genetic architecture of CHH and CDGP to find out a shared genetic basis. This study included 116 CHH probands and 72 CDGP subjects and controls (from ExAC and CoLaus). Exome sequencing showed mutations in IHH genes (25 genes including *IGSF10*) in 51% of CHH subjects, in 7% of CDGP probands and in 18% of controls. Oligogenic inheritance was found in 15% of CHH cases and in only 1.4% of CDGP subjects and 2% of controls, confirming its role in CHH. These results suggest a different genetic architecture of these two conditions; however, considering the role of genes determining CHH in the pathophysiology of pubertal failure, it is possible to hypothesize that, in a small number of individuals with self-limited DP, a pathogenic mutation in one of these genes could be found.

### New Genes Causing Self-Limited Delayed Puberty

Recently, the application of NGS technology to self-limited DP revealed genes involved in determining this phenotype and unraveling interesting scenarios in the genetic control of puberty.

#### HS6ST1

Howard et al. tried to identify genes that are involved in self-limited DP in several studies. In one of them (99), the study population consisted of 492 subjects from the Finnish DP cohort with a diagnosis of self-limited DP as performed in a specialist center from 1982 to 2004. WES was performed on 160 subjects, comprised of 67 probands with DP (57 male and 10 female) from 67 families, 58 affected relatives (36 male and 22 female), and 35 unaffected family members (13 male and 22 female). The results were filtered, giving priority to genes causing HH (28 genes), and identified one variant in the *HS6ST1* gene. Subsequently, target exome sequencing was performed in 288 other individuals from 42 families of the same cohort (178 DP and 110 controls), confirming one pathogenic variant in *HS6ST1* in one family. This was a heterozygous missense variant (p.Arg375His) defined as deleterious by five prediction tools and affecting a highly conserved residue found in a patient with growth delay and with puberty onset at the age of 14.3 years. Other family members with DP were the father, paternal uncle and sister. There was no family history of HH, anosmia was absent in the patient and the relatives with DP.

The identified variant induced a reduced sulfotransferase activity *in vitro*. A murine model was realized. *Hs6st1* mRNA was not expressed in GnRH cells but in regions like the olfactory bulb, in the pre-optical medial area of the hypothalamus and in the arcuate nucleus, which is involved in the regulation of GnRH neurons. The heterozygous murine model allowed the observation of normal localization and number of GnRH neurons in the pre-optical medial area and confirmed the delayed puberty without alteration of body size, testicular structures and adult fertility.

The role of *HS6ST1* gene in hypogonadotropic hypogonadism was investigated in a previous study involving 338 patients with GnRH deficiency (271 males and 67 females), of which 105 have a positive family history, finding a variant in seven subjects corresponding to 2% of IHH patients (100), one homozygous and four heterozygous. In this work, the inheritance pattern was complex, overcoming a simple mendelian transmission, with clinical heterogeneity leading to a hypothesis on the role of epigenetic factors or additional mutations in other genes to fully explain the phenotype.

To understand the role of HS6ST1 in self-limited DP, it is important to consider the biological process of which it takes part. As suggested by the authors (99), in line with *Hs6st1* expression, the reduced sulfotransferase activity in regions including kisspeptin neurons and other cells that influence GnRH function and secretion can impair the overall regulation of GnRH neurons. Furthermore, *Hs6st1* activity is required for the normal function of *Anos1* and *Fgfr1* (100). Therefore, the clinical phenotype of CHH could be due to mutations in the multiple components of this network. In general, it could be plausible that for the same gene a mutation in a single allele can cause self-limited DP. Conversely, a more damaging mutation or the contemporary effect of another gene can lead to a more severe phenotype like CHH. Although a potentially pathogenic variant in *HS6ST1* in self-limited DP has been identified, the absence of mutations in other CHH genes in the analyzed cohort confirms the different genetic basis of CHH and self-limited DP or suggests that other genes causing self-limited DP are yet unknown.

#### IGFS10

This new gene variant was discovered through the application of WES in a cohort of subjects with self-limited DP in trying to explore the genetic basis of this condition. This interesting work was published by Howard et al. (101), in which a cohort of 111 individuals from 18 families (76 DP and 35 controls) was analyzed using WES. They identified two N-terminal variants in *IGSF10* (p.Arg156Leu and p.Glu161Lys) in 20 subjects with self-limited DP from six families, with autosomal dominant mode of inheritance for all except in one patient that presented DP without mutation. They also identified two C-terminal variants (p.Glu2264Gly and p.Asp2614Asn) in the same gene in four other families. In one family, there was an incomplete penetrance, in another, they supposed a *de novo* mutation. All the patients presented a normal growth rate before puberty and a classical DP with delayed pubertal spurt and normal (self-reported) olfaction. The *IGFS10* gene was not previously reported as the cause of human pathology. The murine model allowed the observation that *Igsf10* mRNA is expressed in the nasal mesenchyme of embryos. Additionally, *Igsf10* knockdown disrupts the migration and the neurite elongation of GnRH3 cells *in vivo*. The authors investigated the potential role of *IGF10* in subjects with permanent GnRH deficiency, like in KS, idiopathic hypogonadism and functional HH, like hypothalamic amenorrhea or its equivalent. They performed target exome sequencing on 334 adult patients with KS (162 subjects), IHH (158 subjects), hypothalamic amenorrhea or functional HH (14 subjects). The study described a potentially pathogenic variant in 10.2% of these subjects: 3 loss-of-function variants in 5 patients and 13 missense variants in 29 subjects. Among 14 patients with functional HH, 25% had a personal history of DP and two patients had a heterozygous loss-of-function *IGFS10* variant with no family history of IHH or anosmia and with normal brain MRI. In both cases, an important contemporary environmental factor triggered the functional HH: secondary amenorrhea caused by excessive physical exercise and important weight loss due to a subclinical eating disorder.

This interesting finding suggests a shared etiology between self-limited DP and some forms of functional HH. The functional study performed by the authors revealed that *Igsf10* is involved during the early phase of GnRH neuron migration from the olfactory placode to the hypothalamus and the pre-optic areas. This process has a precise timing and is a pre-requisite for the normal development of the hypothalamic–pituitary–gonadal axis. In case of mutation, the alteration of the *Igsf10* signaling could lead to a reduced number of GnRH neurons that migrate into the hypothalamus or to an incorrect timing, determining puberty onset delay (101).

Considering the role of *IGFS10* in the formation of the GnRH network, it is reasonable to assimilate environmental factors to a “second hit” acting on the hypothalamus–pituitary–gonadal axis made more susceptible to functional hypogonadism by *IGSF10* variants.

#### FTO

Energy homeostasis through the regulation of fat mass and adipokyne production is supposed to be implicated in pubertal timing. In a paper published in 2018 (102), Howard et al. found two rare variants in *FTO* gene in three out of 67 families with self-limited DP. *FTO* gene polymorphisms have been associated both with obesity and with age at menarche.

Of note is that the patients with *FTO* rare variants displayed low body mass index during childhood, suggesting that the effect of that genetic variants occurred through metabolism derangement that in turn can affect puberty onset. The authors also realized a murine model of a heterozygous state of *FTO* that showed delayed puberty (timing of vaginal opening). These two elements support the role of genes involved in the control of energy balance as a possible cause of self-limited DP.

#### EAP1

The same group identified two rare variants in the *EAP1* gene in two families from the same cohort of 67 families with DP (103). *EAP1* contributes to the initiation of female puberty *via* transactivation of the GnRH promoter. *Eap1* is a nuclear transcription factor able to act in two modes: trans-activating the GnRH promoter and by inhibition of the prepoenkephalin promoter, which normally antagonize the GnRH secretion, thus determining an increase of GnRH levels that typically occurs at the start of puberty. Therefore, *EAP1* is an important element of the complex network upstream of GnRH which is involved in the onset of puberty. Mutations in *EAP1* could be a plausible cause of DP, similarly to other genes acting through the alteration of the GnRH secretion (i.e., *KISS1, TAC3* and *TACR3*). However, no mutations that cause disorders of puberty have been identified before of this work. The authors found a rich expression of *Eap1* in the hypothalamus of mouse in peripubertal phase and for *EAP1* mutant proteins showed altered levels and impaired GnRH promoter activity. No phenotypic peculiarity was present in patients with *EAP* variants except for self-limited DP and delayed bone age.

Table 1 summarizes the phenotypic characteristics of patients with a monogenic cause of self-limited DP.

**Table 1:** Patients with monogenic cause of self-limited delayed puberty.

**Gene**	**Number of probands reported (sex)**	**Genetic defects (mutations)**	**Inheritance**	**Pubertal development age at onset (year)**	**Other clinical features**	**Other family members**	**References**
*HS6ST1*	1 (M) from 1 family	Heterozygous missense variant p. Arg375His	Autosomal dominant	14.3	No growth delay before puberty Normal smell (self-reported)	4 (sister, paternal uncle and aunt, father)	(99)
*IGSF10*	10 from 10 families (proband sex 9 M, 1 F)	Heterozygous missense N-terminal variants (p. Arg156Leu; P. Glu161Lys); C-terminal variants (p.Glu2264Gly; p.Asp2614Asn)	Autosomal dominant for N-terminal variants; Autosomal dominant for C-terminal variants but incomplete penetrance in 1 family and a possible *de novo* mutation in another family	13.94-16.5	No growth delay before puberty, normal sense of smell (self-reported)	21 (14 with N-terminal variants from 6 families and 7 with C-terminal variant from 4 families)	(101)
*FTO*	3 (M) from 3 families	Heterozygous missense variants p.Ala163Thr and p.Leu44Val	Autosomal dominant	Not available	Age- and sex-adjusted body mass index in the lower range	11	(102)
*EAP1*	2 (M) from 2 families	Heterozygous in-frame deletion p. Ala221del (family A), heterozygous missense variant p. Asn770His (family B)	Autosomal dominant	Family A proband 15.7 Family B proband 16.5		Family A sister and father Family B mother	(103)

These recent findings suggest that genes determining self-limited delayed puberty could be involved in the overabundant mechanisms that control puberty onset (e.g., modulation of GnRH function and secretion, numerousness of neurons migrating from the olfactory placode to the hypothalamus and the pre-optic areas or energy balance) in spite of genes implicated in CHH that directly affect GnRH neurons migration or function. However, more studies are needed to unveil if this suggestion is correct.

## Conclusions

Delayed puberty is a frequent problem in clinical practice. The most common underlying condition is self-limited DP, but other pathological causes may underlie this condition and should be excluded.

**Figure 1:**
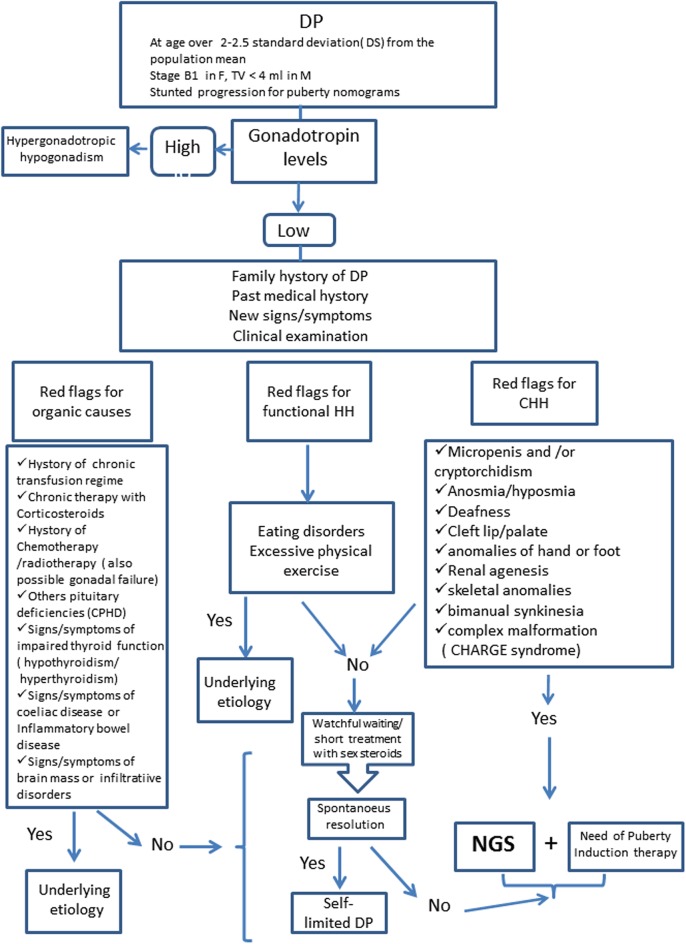
Diagnostic Algorithm for Delayed Puberty. DP, Delayed Puberty; HH, Hypogonadotropic Hypogonadism; CHH, Congenital Hypogonadotropic Hypogonadism; CPHD, Combined Pituitary Hormone Deficiency; NGS, Next Generation Sequencing.

Distinguishing between self-limited DP and permanent HH might be challenging. Observation is appropriate in benign variants of puberty and in those with milder forms of delayed puberty. Genetic testing is appropriate and may be a crucial diagnostic step in cases of DP associated to syndromic features or other red flags to identify IHH patients (see Figure 1). The application of genetic testing in clinical practice for the differentiation of the conditions of self-limited DP and GnRH deficiency would represent a great advantage for diagnosis.

Recent technology advancement (i.e., NGS technology) is allowing the identification of the genetic cause of self-limited delayed puberty as well.

However, as summarized in the present work, patients with monogenic forms of self-limited DP represent a minority of cases and do not have clinical characteristics that allow clinicians to distinguish them.

Furthermore, tests for pathogenicity *in vitro* and *in vivo* and/or assessment of segregation with phenotype within pedigree are needed to demonstrate the causal link between variants found and self-limited DP.

For these reasons, and also for the difficult and challenging interpretation of NGS analysis results (17), we suggest that genetic analysis in patients with self-limited DP be limited to research setting.

However, in the future, as knowledge of the genetic architecture of delayed puberty will be enriched, genetic testing could represent a useful diagnostic tool also in clinical practice.

## Author Contributions

All authors listed have made a substantial, direct and intellectual contribution to the work, and approved it for publication.

## Conflict of Interest

The authors declare that the research was conducted in the absence of any commercial or financial relationships that could be construed as a potential conflict of interest.
